# Reproductive Success of *Trichogramma ostriniae* over *Trichogramma dendrolimi* in Multi-Generational Rearing on Corn Borer Eggs

**DOI:** 10.3390/insects16030297

**Published:** 2025-03-12

**Authors:** Yu Wang, Asim Iqbal, Kanwer Shahzad Ahmed, Yuan-Yuan Zhou, Chen Zhang

**Affiliations:** 1 Agricultural College, Jilin Agricultural Science and Technology University, Jilin 132101, China; wangy19940504@163.com (Y.W.); zhouyy9911@163.com (Y.-Y.Z.); 2 Imdaad: Integrated Facilities Management Company, Street Number 1100, South Zone Jebel Ali, Dubai P.O. Box 18220, United Arab Emirates; asim_iqbal990@yahoo.com; 3 Biological Research & Resource Center, Mastermind Scientific Consultants (SMC-Private) Limited, Sargodha 40100, Punjab, Pakistan; rajput.shahzad18@gmail.com

**Keywords:** Asian corn borer, biological control, emergence rate, offspring mortality, reproductive success, *Trichogramma ostriniae*, *Trichogramma dendrolimi*

## Abstract

In China, the Asian corn borer (ACB), *Ostrinia furnacalis* (Guenee) (Lepidoptera: Pyralidae), is the most significant economic insect pest of corn, causing losses ranging from six to nine million tons annually by feeding on all parts of maize, including damaging ears and leaves and making tunnels in stems. In China, since the 1970s, the *Trichogramma* species have extensively mass-reared from factitious hosts to control ACB and support integrated pest management programs. The *Trichogramma dendrolimi* Matsumura and *T. ostriniae* Pang and Chen are the most efficient biocontrol agents for controlling ACB among the available *Trichogramma* species. To evaluate the reproductive success of *Trichogramma dendrolimi* and *T. ostriniae*, we assessed the impact of varying parasitoid ratios (5:1, 3:1, 1:1, 1:3, and 1:5 representing T. dendrolimi and T. ostriniae, respectively) on adult offspring emergence and mortality from ACB eggs over multiple generations (from first (F_1_) to third (F_3_) generations). We discovered that both *Trichogramma* species’ offspring emergence was significantly influenced by the parasitoid generations, parasitoid ratios, and their interactions. The offspring mortality in both *Trichogramma* species was significantly affected by parasitoid generations but was not significantly influenced by parasitoid ratios or interaction between parasitoid generations and parasitoid ratios. Furthermore, at parasitoid ratios of 5:1, 3:1, and 1:1, the emergence rate of the F_1_ generation of *T. dendrolimi* was significantly higher compared to the ratios of 1:3 and 1:5. However, in the F_2_ generation, the emergence of *T. dendrolimi* decreased considerably, and no emergence was observed in the F_3_ generation. A contrasting trend was observed in the emergence of *T. ostriniae* offspring. Overall, regardless of the parasitoid ratios, the offspring emergence of *T. ostriniae* in all three generations was significantly higher than that of *T. dendrolimi*. After assessing the offspring mortality in our research by dissecting the unhatched eggs, we found an inverse relationship between the *T. dendrolimi* generations and their mortality across different parasitoid ratios. Notably, mortality exhibited a significant decline with an increasing number of generations. A positive correlation was observed between the number of *T. ostriniae* generations and their mortality across different parasitoid ratios, indicating that mortality increased with successive generations. Overall, across all parasitoid ratios and generations, the offspring mortality of *T. ostriniae* was considerably greater than that of *T. dendrolimi*. These results suggest that mortality is a crucial empirical measure that validates *T. ostriniae*’s superiority over *T. dendrolimi*. These findings highlight the importance of selecting suitable parasitoid species when implementing *Trichogramma* for pest management.

## Simple Summary

To evaluate reproductive success, the emergence of adult offsprings of two egg parasitoids, *Trichogramma dendrolimi* Matsumura and *T. ostriniae* Pang and Chen from the host eggs (Asian corn borer, *Ostrinia furnacalis* Guenée), as well as the adult offspring mortality from the unhatched host eggs, was compared under different parasitoid ratios across multiple generations. We discovered that both *Trichogramma* species’ offspring emergence were significantly influenced by the parasitoid generations, parasitoid ratios, and their interactions. The offspring mortality in both *Trichogramma* species was significantly affected by parasitoid generations but was not significantly influenced by parasitoid ratios or interaction between parasitoid generations and parasitoid ratios. Furthermore, across all parasitoid ratios, *T. ostriniae* outcompeted *T. dendrolimi* by F_3_ generation, achieving full emergence while completely suppressing *T. dendrolimi* emergence. Moreover, after assessing the offspring mortality in our research by dissecting the unhatched eggs, we found that, across all parasitoid ratios and generations, the offspring mortality of *T. ostriniae* was considerably greater than that of *T. dendrolimi*. These results suggest that mortality is a crucial empirical measure that validates *T. ostriniae*’s superiority over *T. dendrolimi*.

## 1. Introduction

For more than sixty years, the cultivated corn in the Western Pacific region of Asia was prone to the highly devastating Asian corn borer (ACB), *Ostrinia furnacalis* (Guenee) (Lepidoptera: Pyralidae) [1,2,3]. The distribution of the ACB extends northward across Northern China and eastward from India to Southern China, Japan, Korea, and the Philippines, reaching as far as Australia and the Solomon Islands [1,4,5,6,7]. The ACB larvae have caused more than thirty percent loss of maize products by feeding on all parts of maize, including damaging ears and leaves and making tunnels in stems [1,8,9]. Globally, maize is the leader among grain crops in terms of the highest planting areas [10]. In China, the ACB is the most significant economic insect pest of corn, causing losses ranging from six to nine million tons annually [11,12]. The synthetic insecticides serve as agents to control insect pests, such as ACB [13,14]. The extensive and indiscriminate application of synthetic chemicals negatively affect people’s health and natural environment [13]. Therefore, biological control is a deliberate, promising, and economical approach to managing insect pests worldwide [15].

The members of the family Trichogrammatidae are the most commonly used natural enemies for biological control programs [15]. Currently, Trichogrammatidae comprises about eight hundred species belonging to ninety genera [16]. The *Trichogramma* is the largest genus and contains about two hundred and thirty species [16]. The *Trichogramma dendrolimi* Matsumura and *T. ostriniae* Pang and Chen are the most efficient biocontrol agents for controlling ACB among the available *Trichogramma* species [15]. In China, since the 1970s, the *Trichogramma* species are extensively mass reared from factitious hosts to control ACB [17] and supporting integrated pest management programs [18]. Iqbal et al. [19] revealed that rearing *T. ostriniae* on the factitious host, *Antheraea pernyi* Guerin-Meneville, 1855, via multiparasitism with *Trichogramma chilonis* Ishii facilitates enhanced biocontrol potential against ACB. Furthermore, the effect of various parasitoid ratios on the numeric response of the *Trichogramma* species is indeed also crucial for optimizing their role in biological control programs. Ghaemmaghami et al. [20] observed the changes in functional and numerical responses of the parasitoid wasp, *Trichogramma brassicae* Bezdenko, 1968, over forty-five generations of rearing on *Ephestia kuehniella* Zeller, 1879. The parasitism and offspring’s emergence are the key functional and numerical responses that determine the fate of the effectiveness of parasitoid mass-rearing systems [20,21,22]. Regarding the importance of multiparasitism in parasitoid mass-rearing, Iqbal et al. [23] revealed that the optimal offspring emergence from one host egg (Chinese oak silkworm, *Antheraea pernyi* Guerin-Meneville) was observed when the *T. ostriniae* were in multiparasitism with *T. chilonis*. In China, *T. chilonis*, *T. dendrolimi*, and *T. ostriniae* are efficiently mass reproduced on *A. pernyi* through monoparasitism [24]. Therefore, more research is needed to explore the performance of *T. ostriniae* on ACB eggs and investigate the offspring’s emergence through multiparasitism along with *T. dendrolimi.*

Reproductive success plays a pivotal role in understanding the dynamics of multiparasitism in *Trichogramma* species, influencing both their competitive interactions and effectiveness in biological control [25]. Li et al. [26] revealed that *T. dendrolimi* showed reproductive success over *T. ostriniae* due to more adults emerging from *A. pernyi* eggs under a multiparasitism regime. Moreover, *T. dendrolimi* played a pivotal role in helping the optimal emergence of *T. ostriniae* offsprings because *T. ostriniae* offsprings failed to make enough holes in the host egg for their emergence due to the hard chorion of *A. pernyi* egg; therefore, *T. ostriniae* utilized the hole created by the *T. dendrolimi* for the exit [26]. More research is required to investigate the reproductive efficiency of these two parasitoid species at varying ratios while rearing on ACB eggs. Therefore, in the present study, we evaluated the offspring emergence and mortality of *T. dendrolimi* and *T. ostriniae* from ACB eggs at multiple generations under laboratory conditions to provide insights into using *Trichogramma* species for biological control of the Asian corn borer, making pest management more efficient, cost-effective, and environmentally sustainable.

## 2. Materials and Methods

### 2.1. Parasitoids

In 2023, the adult parasitoids *T. dendrolimi* and *T. ostriniae* were obtained from the parasitized ACB eggs in the corn fields of Changchun, Jilin province, China (43.89° N, 125.32° E). Based on the morphological characteristics of the male genitalia, both parasitoid species were identified using scanning electron microscope micrographs [27], and rDNA ITS2 sequence analysis confirmed this identification [28]. In the laboratory, these parasitoids were cultured on ACB eggs for five generations in an incubator (MLR-351H; Sanyo Corporation, Moriguchi, Osaka, Japan) under optimum conditions (L14: D10, 26 ± 1 °C, 65 ± 5% RH).

### 2.2. Host

#### Asian Corn Borer (ACB), *Ostrinia furnacalis*

ACB pupae were maintained in a cage (35 × 35 × 35 cm; Bugdorm-I, Taichung, Taiwan, China) at feasible conditions (L14: D10, 26 ± 1 °C, 65 ± 5% RH) to collect eggs for experimentation. After the emergence, the moths were provided with a 20% honey solution as a sustainable food source, and a large piece (30 cm × 30 cm) of wax paper was suspended in the cage for oviposition. After oviposition, the part of the wax paper containing egg masses was cut with a scissor and kept in a climate chamber room (Faithful Instrument Co., Ltd., Huanghua, Hebei, China) under optimal conditions (L14: D10, 26 ± 1 °C, 65 ± 5% RH) until they attained the age of less than 4 h for experimentation.

### 2.3. Comparative Reproductive Success of Trichogramma Species Across Generations

To assess the reproductive success between *T. dendrolimi* and *T. ostriniae*, the ACB eggs (aged < 4 h) were parasitized under controlled environmental conditions (L14: D10, 26 ± 1 °C, 65 ± 5% RH). For this purpose, the wax paper containing 70 ACB eggs was stapled to the dorsal leaf surface of a corn plant placed in a cage (2 × 2 × 2 m; Bugdorm-I, Taichung, Taiwan, China). Subsequently, each mated adult female of *T. dendrolimi* and *T. ostriniae* that had newly emerged (<12 h prior) was introduced into the cage containing ACB eggs for parasitization. Additionally, more diverse ratios of *T. dendrolimi* and *T. ostriniae* (3:1, 5:1, 1:3, and 1:5) were examined in four separate cages containing ACB eggs with a corn plant. After 8 days of parasitism, the parasitized eggs from all the cages were transferred to individual glass tubes (10 × 3 cm, length × diameter; Shanghai Allcan Medical Co., Ltd., Pudong New Area, Shanghai, China) and placed in an incubator under controlled conditions (L14: D10, 26 ± 1 °C, 65 ± 5% RH) for development. The emergence of the first generation (F_1_) of both wasps was monitored daily, and cotton-soaked 20% honey solution was provided as adult food. Furthermore, the number of adult offspring was recorded until complete emergence. The *T. dendrolimi* and *T. ostriniae* were identified with the help of morphological descriptions provided by Myint et al. [29]. The unhatched eggs were dissected to ascertain the offspring’s mortality. All treatments were replicated ten times. Furthermore, all the emerged offspring of the F_1_ generation from a single treatment were transferred to a cage containing 30 masses of ACB eggs (each mass contains 70 eggs) (aged < 4 h) stapled with the leaf surface of a corn plant. After 8 days of parasitism, 15 randomly selected parasitized egg masses were placed in a glass tube (10 × 3 cm, length × diameter; Shanghai Allcan Medical Co., Ltd., Pudong New Area, Shanghai, China) and then kept in an incubator under controlled conditions (L14: D10, 26 ± 1 °C, 65 ± 5% RH) for development. After the emergence of second generation (F_2_), the species were identified based on the abovementioned procedure, and both wasps’ emergence rate and mortality were recorded. The same experimental procedure was adopted to evaluate the emergence rate and mortality of offspring of the third generation (F_3_).

The offspring emergence rate (%) for *T. dendrolimi* and *T. ostriniae* was calculated based on the following formula:

$$\begin{array}{l} Td\;offspring\;emergence\;rate\left(\%\right)\\ \;=Number\;of\;emerged\;Td/(Number\;of\;emerged\;Td\\ \;+Number\;of\;emerged\;To)\times 100 \end{array}$$

$$\begin{array}{l} To\;off\;spring\;emergence\;rate\left(\%\right)\\ \;=Number\;of\;emerged\;To/(Number\;of\;emerged\;Td\\ \;+Number\;of\;emerged\;To)\times 100 \end{array}$$

The offspring mortality (%) for *T. dendrolimi* and *T. ostriniae* was calculated based on the following formula:

$$\begin{array}{l} Td\;off\;spring\;mortality\left(\%\right)\\ \;=Number\;of\;dead\;adult\;Td/(Number\;of\;dead\;adult\;Td\\ \;+Number\;of\;dead\;adult\;To)\times 100 \end{array}$$

$$\begin{array}{l} To\;offspring\;mortality\left(\%\right)\\ \;=Number\;ofdead\;adult\;To/(Number\;of\;dead\;adult\;Td\\ \;+Number\;of\;dead\;adult\;To)\times 100 \end{array}$$

### 2.4. Statistical Analysis

Data were analyzed by the statistical software IBM SPSS^®^ (Statistical Package for Social Science) v8.0. The data regarding offspring emergence and mortality were analyzed using factorial analysis of variance (ANOVA), and treatment means were further compared by Tukey’s honestly significant difference (HSD) test at a 95% confidence interval. Prior to analysis, data were normalized by arcsine square root transformation (arsin (sqrt(x))). Data were displayed using SigmaPlot 12.5.

## 3. Results

### 3.1. Effect of Parasitoid Ratio Variations on Offspring Emergence Across Generations in Trichogramma Species

**Figure 1:**
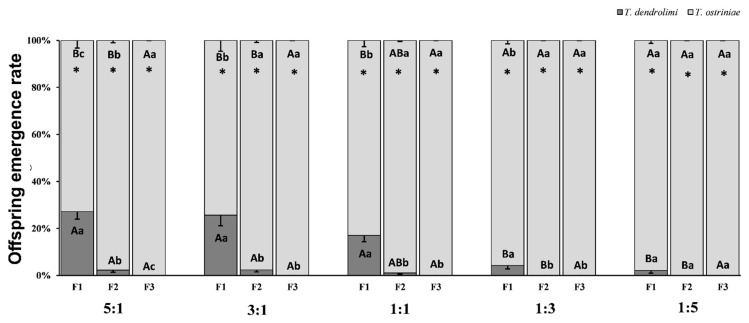
Percentage of (mean ± SE) emerged offspring of *Trichogramma dendrolimi* and *Trichogramma ostriniae* from *Ostrinia furnacalis* eggs across first (F_1_) to third (F_3_) generations under different parasitoid ratios. The different ratios of *T. dendrolimi* and *T. ostriniae* represent 5:1, 3:1, 1:1, 1:3, and 1:5. Different upper-case letters on the same patterned bars indicate significant differences in offspring emergence rate of *T. dendrolimi* and *T. ostriniae* under different parasitoid ratios, while different lower-case letters on the bars within a given group indicate significant differences in offspring emergence rate of *T. dendrolimi* and *T. ostriniae* between different generations (Tukey’s HSD test, *p* < 0.05). The paired bars with asterisk indicate significant difference in means (*p* < 0.05).

The results showed that the offspring emergence rate of both *Trichogramma* species was significantly affected by parasitoid generations (*p* < 0.0001), parasitoid ratios (*p* < 0.0001), and parasitoid generations × parasitoid ratios (*p* < 0.0001) (Table 1). In the F_1_ and F_2_ generations, there was a significant difference in *T. dendrolimi* emergence among different parasitoid ratios (F_1_:F_4,45_ = 23.592, *p* < 0.0001; F_2_:F_4,45_ = 4.518, *p* = 0.0037). Furthermore, under the parasitoid ratios of 5:1, 3:1, and 1:1, the emergence of F_1_ generation of *T. dendrolimi* was significantly higher (27.2%, 25.7%, and 17%, respectively) than the parasitoid ratios of 1:3 (4.2%) and 1:5 (2.1%), respectively. Comparatively, in the F_2_ generation, the emergence of *T. dendrolimi* was significantly reduced by 91.54%, 90.66%, and 93.52% under parasitoid ratios of 5:1, 3:1, and 1:1, respectively. However, no emergence of *T. dendrolimi* was observed under 1:3 and 1:5 ratios. Moreover, under all parasitoid ratios, no emergence of F_3_ generation of *T. dendrolimi* was observed (Figure 1).

In the F_1_ and F_2_ generations, there was a significant difference in *T. ostriniae* emergence among different parasitoid ratios (F_1_:F_4,45_ = 23.592, *p* < 0.0001; F_2_:F_4,45_ = 4.518, *p* = 0.0037). Furthermore, under the parasitoid ratios of 1:5, and 1:3, the emergence of F_1_ generation of *T. ostriniae* was significantly higher (97.9%, and 95.8%, respectively) than the parasitoid ratios of 1:1 (83%) and 3:1 (74.3%), and 5:1 (72.8%), respectively. Comparatively, in the F_2_ generation, the emergence of *T. ostriniae* was significantly increased by 2.14%, and 4.38%, 19.15%, 31.35% and 34.20% under parasitoid ratios of 1:5, 1:3, 1:1, 3:1 and 5:1, respectively. Moreover, under all parasitoid ratios, complete emergence of F_3_ generation of *T. ostriniae* was observed (Figure 1).

Under the same parasitoid ratios, we compared the *T. dendrolimi* emergence among different generations and found significant differences (5:1:F_2,27_ = 92.828, *p* < 0.0001, 3:1:F_2,27_ = 50.765, *p* < 0.0001, 1:1:F_2,27_ = 76.300, *p* < 0.0001, 1:3:F_2,27_ = 11.780, *p* = 0.0002), indicating that as the number of generations increased, the offspring emergence was gradually decreased. However, under the 1:5 parasitoid ratios, there is no significant difference among various generations for the *T. dendrolimi* emergence (F_2,27_ = 3.724, *p* = 0.0563). The complete disappearance of *T. dendrolimi* progeny was exhibited by an F_3_ generation in 5:1, 3:1, and 1:1 ratio, whereas under 1:3 and 1:5 ratios, no emergence of *T. dendrolimi* progeny occurred in the F_2_ generation (Figure 1).

Similarly, under the same parasitoid ratios, we compared the *T. ostriniae* emergence among different generations and found significant differences (5:1:F_2,27_ = 92.828, *p* < 0.0001, 3:1:F_2,27_ = 50.765, *p* < 0.0001, 1:1:F_2,27_ = 76.300, *p* < 0.0001, 1:3:F_2,27_ = 11.780, *p* = 0.0002). However, unlike *T. dendrolimi*, a contradictory trend was observed, indicating that offspring emergence significantly increased as the number of generations increased. Nevertheless, under the 1:5 parasitoid ratios, there is no significant difference among various generations for the *T. ostriniae* emergence (F_2,27_ = 3.724, *p* = 0.0563). The F_3_ generation exhibited a complete emergence of *T. ostriniae* under the 5:1, 3:1, and 1:1 parasitoid ratios, whereas under 1:3 and 1:5 ratios, complete emergence of *T. ostriniae* progeny occurred in the F_2_ generation (Figure 1). Regardless of the parasitoid ratios, the offspring emergence of *T. ostriniae* in all three generations was significantly higher than that of *T. dendrolimi* (All *p* < 0.05) (Figure 1).

### 3.2. Effect of Parasitoid Ratio Variations on Offspring Mortality Across Generations in Trichogramma Species

**Figure 2:**
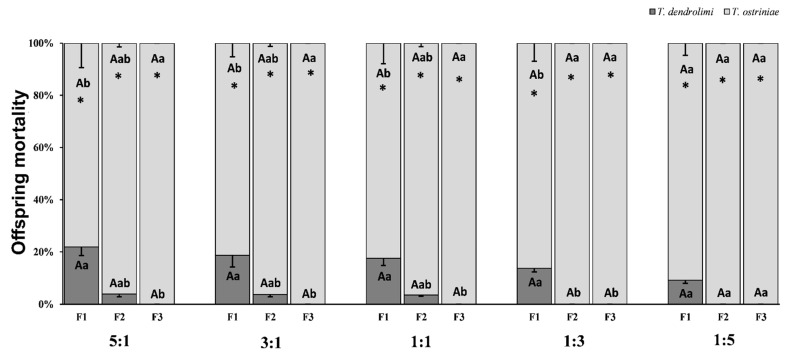
Percentage of (mean ± SE) dead offspring of *Trichogramma dendrolimi* and *Trichogramma ostriniae* from *Ostrinia furnacalis* eggs across first (F_1_) to third (F_3_) generations under different parasitoid ratios. The different ratios of *T. dendrolimi* and *T. ostriniae* represent 5:1, 3:1, 1:1, 1:3, and 1:5. Different upper-case letters on the same patterned bars indicate significant differences in offspring mortality of *T. dendrolimi* and *T. ostriniae* under different parasitoid ratios, while different lower-case letters on the bars within a given group indicate significant differences in offspring mortality of *T. dendrolimi* and *T. ostriniae* between different generations (Tukey’s HSD test, *p* < 0.05). The paired bars with asterisk indicate significant difference in means (*p* < 0.05).

After assessing the offspring mortality in our research by dissecting the unhatched eggs, we found that (based on ANOVA) the offspring mortality in both *Trichogramma* species was significantly affected by parasitoid generations (*p* < 0.0001) but was not significantly affected by parasitoid ratios (*p* = 0.3855), or interaction between parasitoid generations and parasitoid ratios (*p* = 0.9050) (Table 2). In the F_1_, F_2_, and F_3_ generations, there was not a significant difference in *T. dendrolimi* mortality among different parasitoid ratios (F_1_:F_4,42_ = 0.495, *p* = 0.7392; F_2_:F_4,42_ = 3.882, *p* = 0.0881, F_3_:F_4,42_ = 3.882, *p* = 0.0881). The same trend was observed for offspring mortality of *T. ostriniae* in all three generations (F_1_:F_4,42_ = 0.495, *p* = 0.7392; F_2_:F_4,42_ = 3.882, *p* = 0.0881, F_3_:F_4,42_ = 3.882, *p* = 0.0881). Furthermore, under the parasitoid ratios of 1:3, and 1:5, there was no existence of *T. dendrolimi* in F_2_ and F_3_ generations (Figure 2).

Under the 5:1, 3:1, 1:1, and 1:3 parasitoid ratios, we compared the *T. dendrolimi* mortality among different generations and found significant differences (5:1:F_2,25_ = 5.358, *p* = 0.0116, 3:1:F_2,27_ = 8.229, *p* = 0.0016, 1:1:F_2,27_ = 3.636, *p* = 0.0400, 1:3:F_2,26_ = 4.416, *p* = 0.0223), indicating that offspring mortality significantly decreased as the number of generations increased. Additionally, no significant difference was observed between F_1_ and F_2_; however, a significant difference was noted in the F_3_ generation regarding *T. dendrolimi* mortality under the 5:1, 3:1, and 1:1 parasitoid ratios. In the 1:3 and 1:5 ratios, the *T. dendrolimi* mortality in the F_1_ generation was higher than F_2_ and F_3_ generations (Figure 2).

Similarly, significant differences were observed in *T. ostriniae* mortality under the parasitoid ratios 5:1, 3:1, 1:1, and 1:3 (5: 1:F_2,25_ = 5.358, *p* = 0.0116, 3: 1:F_2,27_ = 8.229, *p* = 0.0016, 1:1:F_2,27_ = 3.636, *p* = 0.0400, 1:3:F_2,26_ = 4.416, *p* = 0.0223). Though, dissimilar to *T. dendrolimi*, a contrary trend was observed, indicating that offspring mortality of *T. ostriniae* significantly increased as the number of generations increased. Also, when the parasitoid ratios were 5:1, 3:1, and 1:1, there was no significant difference in the offspring mortality of *T. ostriniae* between the F_1_ and F_2_ generations, but there was a considerable difference compared to the F_3_ generation. In the 1:3 and 1:5 ratios, the mortality of *T. ostriniae* offspring in the F_1_ generation is higher than in the F_2_ and F_3_ generations. Overall, the mortality of *T. ostriniae* offspring was significantly higher than that of *T. dendrolimi* (All *p* < 0.05) in all parasitoid ratios and generations (Figure 2).

## 4. Discussion

Parasitoids are considered long-term, cost-effective and sustainable solutions to manage insect pests in agriculture [30]. Studies on the performance of parasitoid *T. ostriniae* on ACB eggs and investigating the offspring’s emergence through multiparasitism along with *T. dendrolimi* are needed. To better understand the dynamics of multiparasitism, reproductive success plays a key role in analyzing the effectiveness of *Trichogramma* in a biological control program [25]. Our research found significant reproductive success of *T. ostriniae* over *T. dendrolimi*. Furthermore, the offspring emergence and mortality of two *Trichogramma* parasitoid species (*T. dendrolimi* and *T. ostriniae*) significantly differed across multiple generations based on parasitoid ratios for the ACB host. The combine and multiple release of two *Trichogramma* species is a promising biocontrol strategy to control insect pests in the field [31,32]. Sigsgaard et al. [33] evaluated the mix of two *Trichogramma* species, *T. evanescens* and *T. cacoeciae* (1:1), to control *Cydia pomonella* in apple orchards and revealed a 54% reduction in fruit damage in the fields. However, the scientists did not identify the most promising and potential biocontrol candidate between *T. evanescens* and *T. cacoeciae* to control *C. pomonella* eggs. Moreover, they only utilized the same ratio of both *Trichogramma* species. However, in our research, we used various ratios of *T. dendrolimi* and *T. ostriniae* to parasitize ACB eggs and estimated the offspring emergence and mortality across three generations. The results indicated that the highest emergence of the F_1_ generation of *T. dendrolimi* was observed when five *T. dendrolimi* and one *T. ostriniae* were reared on ACB eggs. Apart from the higher density of *T. dendrolimi* (five female parasitoids) concerning *T. ostriniae* (a single female parasitoid), *T. dendrolimi* had better temperature and humidity adaptation than other *Trichogrmma* species, making *T. dendrolimi* a potential candidate for controlling insect pests [34]. Unfortunately, *T. ostriniae* put an evil eye on the reproductive success of *T. dendrolimi* because, at the same parasitoid ratios (5:1) as the generation increase from F_1_ to F_2_, and F_2_ to F_3_, the offspring emergence of *T. dendrolimi* was reduced by 91.54% and 100%, respectively. The *T. ostriniae* is accountable for this sudden reduction in *T. dendrolimi* offspring emergence because the former efficiently parasitized the ACB eggs irrespective of host age, whereas older host eggs are unsuitable for *T. dendrolimi* [35,36]. Moreover, *T. ostriniae* prefers to oviposit the yellow host (ACB egg) compared to *T. dendrolimi* [37]. Additionally, parasitoids may cause egg mortality by multiple drilling without laying eggs in the host [38]. Therefore, the offspring emergence of *T. dendrolimi* was gradually suppressed. Furthermore, the highest emergence of the F_1_ generation of *T. ostriniae* was observed when one *T. dendrolimi* and five *T. ostriniae* were reared on ACB eggs. Therefore, due to its higher density, *T. ostriniae* benefited from *T. dendrolimi* during interspecific competition [39]. Additionally, *T. ostriniae* developed significantly faster at every age of ACB eggs than *T. dendrolimi* [35]. Therefore, *T. ostriniae* outperformed *T. dendrolimi* [40]. Finally, the emergence of *T. ostriniae* progeny increased with the number of generations. Thus, the population growth rate will serve as an adequate indicator of parasitoid performance on different hosts [41]. In addition to progeny emergence, understanding parasitoid mortality is crucial for successful insect pest management [42]. Additionally, mortality is arguably the most widespread empirical measure in entomology [43]. After assessing the offspring mortality in our research by dissecting the unhatched eggs, we found that, across all parasitoid ratios and generations, the offspring mortality of *T. ostriniae* was considerably greater than that of *T. dendrolimi* (All *p* < 0.05). These results suggest that mortality is a crucial empirical measure that validates *T. ostriniae*’s superiority over *T. dendrolimi*. Moreover, Wang et al. [35] also revealed that *T. ostriniae* is more effective than *T. dendrolimi* as a biocontrol agent of the ACB eggs. These findings highlight the importance of selecting suitable parasitoid species when implementing *Trichogramma* for pest management. Further research should build upon these semi-field observations by examining conditions where additional ecological factors may influence parasitoid competition. A comprehensive understanding of parasitoid interactions during multi-generational rearing on ACB eggs will enhance the effectiveness of biological control programs targeting lepidopteran pests.

## 5. Conclusions

The research results indicate that parasitoid generations and ratios affect the emergence and mortality of *T. dendrolimi* and *T. ostriniae* offspring. Due to the complete emergence of *T. ostriniae* in the F_3_ generations, *T. ostriniae* outperformed *T. dendrolimi* regardless of the parasitoid ratios. Additionally, after dissecting the unhatched eggs, the dead offspring was detected. We found that the progeny mortality of *T. ostriniae* was significantly higher than that of *T. dendrolimi* across all parasitoid ratios and generations, indicating that *T. ostriniae* was more prevalent than *T. dendrolimi*. According to these findings, *T. ostriniae* performs the best and is the most viable option for biologically controlling ACB in the fields, eventually increasing maize productivity.

## Figures and Tables

**Table 1:** The effect of parasitoid generations, parasitoid ratios, and parasitoid generations × parasitoid ratios on offspring emergence rate of both *Trichogramma* species from *Ostrinia furnacalis* eggs.

Parameter	Source	df	*Trichogramma* Species	
			F-Value	*p*-Value
Offspring emergence rate (%)	Parasitoid generations	2	1101.536	<0.0001
	Parasitoid ratios	4	603.500	<0.0001
	Parasitoid generations × Parasitoid ratios	8	475.290	<0.0001
	Error	135		

*p* < 0.001 is considered significant; two-factorial ANOVA α at = 0.05.

**Table 2:** The effect of parasitoid generations, parasitoid ratios, and parasitoid generations × parasitoid ratios on offspring mortality of both *Trichogramma* species from *Ostrinia furnacalis* eggs.

Parameter	Source	df	*Trichogramma* Species	
			F-Value	*p*-Value
Offspring mortality (%)	Parasitoid generations	2	25.609	<0.0001
	Parasitoid ratios	4	1.047	0.3855
	Parasitoid generations × Parasitoid ratios	8	0.424	0.9050
	Error	135		

*p* < 0.001 is considered significant; two-factorial ANOVA α at = 0.05.

## Data Availability

The raw data supporting the conclusions of this article will be made available by the authors upon request.
